# Predictive Ability of Future Anxiety in Professional Decision-Making Skill among a Syrian Refugee Adolescent in Jordan

**DOI:** 10.1155/2020/4959785

**Published:** 2020-03-28

**Authors:** Ola Al Hwayan

**Affiliations:** School of Education Science, Department of Counseling and Special Education, University of Jordan, Amman, Jordan

## Abstract

This study aimed to examine how and to what extent future anxiety contributes to the level of professional decision-making skills among Syrian refugee students in the tenth grade in public schools in Jordan for the year 2018/2019. Using a quantitative approach, 227 tenth-grade Syrian refugee students in public schools in the University Brigade Institute of the Ministry of Education in Jordan were measured on scales of future anxiety and professional decision-making. The results showed that the level of future anxiety was high, while the level of professional decision-making was moderate. In addition, the results showed that there were statistically significant differences in the future anxiety depending on gender (i.e., male/female) and achievement level (i.e., high achievement/low achievement), whereas for professional decision-making, there were differences depending on gender but not achievement level. Finally, it showed that future anxiety is predictive of professional decision-making skills.

## 1. Introduction

War represents the most severe crisis and disaster that people experience, and its effects on families within conflict zones can last for many years. Families seeking to live in a new way or in a new society must continue to find ways to cope with the tragic situations they have experienced, which impart severe physical and psychological effects. Adolescents who have experienced aggression, war, immigration, asylum, and other threatening situations may develop behavioural and psychological problems because of physical and psychological injury. These problems include, but are not limited to, symptoms of anxiety, fear, intolerance, inability, social isolation, depression, behavioural problems, and social problems [1, 2].

The crisis of Syrian refugees is spreading, and many asylum seekers remain in Jordan. It is common for Syrian adolescents who fled conflict in their country to have faced exposure to psychological pressure because of the war. That calls for further research on the impact of asylum on mental health, life control, and decision-making, which are central to determining one's career in the fear of unknown and anxiety, confusion, and ambiguity from making decisions among adolescence.

The decision-making process is the cornerstone of most matters of contemporary life because it has implications for the lives of individuals and communities. The decision-making process is a complex intellectual, psychological, and behavioural process that involves gathering information on possible alternatives or solutions to a problem and then choosing the appropriate alternative to reach the desired goal [3].

The future is often affected by sociocultural factors, such as the situation in one's community. The absence of a real, effective, active career may provoke fear and anxiety about the future, and the future may become a source of anxiety as a result of the misperception of potential events. From there, a young person's distrust of his or her ability to deal with these events and a tendency to view the future in a negative way can create difficulty in decision-making [4].

Adolescence is a critical stage in an individual's life, and it is full of problems that cause anxiety. An individual in this stage may be developing different aspects of his or her personality, and he or she may face psychological pressures such as making important life decisions in the face of the surrounding pressures of his or her environment [5]. Future anxiety can be defined as a state of insecurity, fear, and tension that arises from thoughts about changes that are not desirable in the future, and in the most severe cases of future anxiety, the person suffering from anxiety may feel threatened that something that is not real will happen [6].

The choices of school students about university specializations are often based on many changeable factors, including economic, social, and environmental factors, and the interactions of these factors affect students behaviours and lead them to feel nervous and anxious as a result of the contradictions between what is imagined versus what is real, and their dreams versus their future expectations [7].

There are differences between the males and the females in general; in this issue, Anastasi [8] points that there are differences due to gender in many tests, and the males and females achieve different results in personal, ability, tendencies, and attitude tests. Furthermore, research has shown that there are differences due to gender for future anxiety [9–11] and decision-making [12–14].

To address this research gap, the present study looks at this topic with a focus on the future anxiety due to the traumatic experiences that accompany displacement and asylum. The new, unexpected phase imposed on them as refugees and the unfamiliar environmental, social, and psychological experiences can all influence their professional decision-making. The associated decisions will have a long-term impact on future lives.

### 1.1. Research Questions

1. What is the level of the future anxiety and the professional decision-making skills?
2. Do future anxiety and professional decision-making vary with gender and/or academic achievement?
3. Does future anxiety predict professional decision-making skill?

## 2. Method

### 2.1. Research Design

This study uses quantitative to investigate the levels of the future anxiety and professional decision-making and the predictive ability of future anxiety in professional decision-making skills among Syrian refugee adolescents in public schools Jordan.

### 2.2. Participants

All of the tenth-grade Syrian refugees in public schools at the University Brigade Institute of the Ministry of Education in Jordan participated in this study (227 participants, distributed across seven schools). Five of the schools that participated were for boys (134 male students), and three of the schools were for girls (93 female students).

### 2.3. Data Collection Tools

#### 2.3.1. Future Anxiety Measure

The researcher adapted the measure from Al-Sharifen, Bani-Mostafa & Tashtosh [11]; Salme [15]; and AlMomani & Na'em [16]. This tool comprises 34 items that measure future anxiety. The researcher developed this measurement because there was no existing tool developed for Syrian refugees. The measurement rated each item on a five-point scale, from 1 (“never applies”) to 5 (“applies greatly”). Higher scores reflect a higher level of future anxiety. The researcher in this study extracted the validity and reliability, like context validity by reviewing it from 10 specialists in the universities, and it is applied on a pilot study to examine the internal validity correlations ranged between 0.30 and 0.58, while its acceptable value indicated to [17]. The internal consistency of the initial test was 0.87, while the retest two weeks later had an internal consistency with Cronbach alpha test of 0.85, and all were significant at *p* < 0.05. The range of possible raw scores on the scale is 34–170, and future anxiety is considered high for scores from 137 to 170. Scores 136–103 indicate moderate future anxiety, and scores of 34–102 indicate low future anxiety.

#### 2.3.2. Professional Decision-Making Measure

The researchers conducting this study adapted the measure from AbuNjeleh [18] and Bani Fawaz [19]. This tool comprises 30 items that measure professional decision-making abilities. The researcher developed this measurement because there was no measure developed for Syrian refugees. Participants were required to rate each item on a five-point scale, from 1 (never applies) to 5 (applies greatly), and higher scores on the measure reflect a higher level of professional decision-making. The researcher in this study extracted the validity and reliability, like context validity by reviewing it from 10 specialists in the universities, and it is applied on a pilot study to examine the internal validity correlations ranged between 0.39 and 0.83, while its acceptable value indicated to Kaplen & Saccuzzo [17]. The internal consistency with Cronbach alpha test was 0.90 on the initial test and 0.87 for the retest taken two weeks later; all were significant at *p* < 0.05. The range of raw scores on the total scale is 30–150; the level of professional decision-making is high for scores of 111–150, moderate for scores of 110–71, and low for scores of 30–70.

### 2.4. Data Collection

The researcher obtained approval for this study from the Institutional Review Board in the Jordan Ministry of Education. Data was collected from tenth-grade Syrian refugee students in public schools in the University Brigade Institute of the Ministry of Education in Jordan during regular class time with a help from the school counsellors. The school administration gave approval for both measures (i.e., the future anxiety and professional decision-making tools).

### 2.5. Data Analysis

The data were analysed using SPSS. For Research Question 1, the means and standard deviations were calculated for the two measures: future anxiety and professional decision-making, while for the second question, MANOVA was used. Finally, for the last question, a simple linear regression analysis was conducted.

## 3. Findings

### 3.1. What Is the Level of Future Anxiety and Professional Decision-Making Skills among Tenth-Grade Syrian Refugees?

The means and standard deviations of the data were calculated for professional decision-making and future anxiety measures.  Table 1 displays the results.

Table 1 shows that the dimension averages of the professional decision-making skills ranged from 2.76 to 2.86, which reflects a moderate level in these skills, while the total average of the professional decision-making skill was also moderate at 2.83. The average of the future anxiety was high (3.52).

The high level of future anxiety can be attributed to the harsh and difficult conditions experienced by refugees, such as violence, murder and loss of family and friends, being forced to migrate from the homeland, and living in refugee camps. The need to leave the camps in search of a better life is also a great challenge, and collectively, these experiences lead to instability and fear of future events.

The moderate level of professional decision-making could be attributed to the hesitation and perplexity that the refugee adolescents feel towards their past dreams and ambitions, as they face a difficult reality with demotivating political, social, economic, and even family factors. They may struggle to choose between what they want and what reality and external forces impose on them.

This result reflects a marked decline in decision-making skills when compared to the results of the same age group of Jordanian children, as in the Al-Fuaaa study (2017), the Rashidi study (2017), and the Safouri study (2014) at a high level.

### 3.2. Do Future Anxiety and Professional Decision-Making Vary with Gender and/or Academic Achievement?

The averages and standard deviations were calculated for future anxiety and professional decision-making skill scores according to gender and academic achievement.  Table 2 displays the results.

Table 2 shows that there are apparent differences in averages in both the future anxiety measure and the professional decision-making measure according to gender and academic achievement. To find out if these differences were statistically significant, MANOVA was used as shown in Table 3.

Table 3 indicates that there are statistically significant differences (*p* = 0.05) in the levels of future anxiety and the professional decision-making according to the gender variable, with males in future anxiety and with females in professional decision-making. There are also differences in levels of the professional decision-making according to the academic achievement with weak achievement by Schefee test; however, there are no statistically significant differences in the level of future anxiety according to academic achievement.

The decision-making skill among females is higher than for males because the choices available to the females in a foreign country can be limited due to the conservative culture prevailing in a closed society such as refugee communities. There are often negative attitudes that parents carry towards girls' education after the basic stage, and trends lean towards early marriage of women as they are a burden on their families. Therefore, women do not have the right to self-determination, and there are men who make decisions about them. The result of this study is consistent with Al-Barashdiya [20].

The higher level of future anxiety among men is consistent with the study of Al-Talaa [21], who attributed this to the fact that in Eastern societies, responsibility in the family belongs to men. Therefore, men tend to feel committed and responsible for their families and for providing them with a financial income that will help them to live. This added pressure can cause high levels of anxiety among men.

The results of the professional decision-making skill can be attributed to the fact that low-achieving students do not hesitate much to make his professional decision and make them quickly, given that they already know that they will be unable to complete university education because of weak achievement. Most refugee students have weak achievement at school; many do not complete their studies after the tenth grade, and they go on to work in professional or businesses industries that do not require a high degree. Yet, those with moderate and high achievement suffer from internal conflict and sorrow from the reality they cannot change; they carry ambitions and great dreams about completing their studies, but they may be shocked when they find themselves face-to-face with a difficult reality, such as their family being unable to help them materially. Therefore, when it comes to professional decision-making and determining one's future academic course, choices are not only based on abilities and inclinations, but also on external factors that will affect one's decision.

### 3.3. What Is the Predictive Ability of Future Anxiety, Gender, and Achievement Level on Professional Decision-Making Skill among a Syrian Refugee Adolescents in Jordan?

To find out the impact of future anxiety, gender, and achievement on the professional decision-making skills among Syrian refugee adolescent students, a simple linear regression analysis was calculated, and Table 4 shows the results.

Table 4 indicates an effect of the variables (i.e., future anxiety, gender, and achievement level) on professional decision-making skill, as shown by the *F* value of 23.959, which was statistically significant at level of (*p* < 0.05). The *R*^2^ value indicates that the future anxiety explains 33.1% of changes in the level of professional decision-making. The relationships between future anxiety and decision-making and achievement were negative.

This study is the first of its kind in relation to the variables and that there have been no previous studies that correlate the future anxiety and the professional decision-making of Syrian refugees.

The psychological state of the individual is one of the most important factors affecting human behavior, as hyper-anxiety affects on feelings and emotions also extends to the individual ability to the right psychological balance, which in turn affects the individual behavior in general, and the decision-making process is considered one of the most important requirements. The adolescent's experience affects his or her career, so the decision-making process needs a personal and psychological compatibility. Because of the difficult circumstances and fear of the unknown experienced by refugees, this balance is negatively affected, which negatively affects the professional decision-making process.

Achievement is considered an important factor in professional decision-making and plays an important role in the student's educational and professional decision, as many educational institutions adopt percentages of student grades in the university admission process, which in turn makes achievement relevant in the professional selection process. Despite the negatives of this method in the selection process, it is not an honest indicator that can predict a successful future for the student, so we find that many students choose to make choices that are appropriate to their score totals and not based on their desires, inclinations, and real abilities. As for the specificity of the sample study and the circumstances of the refugees, as we mentioned earlier that the low-achieving person does not hesitate much in making his professional decision and makes his decision quickly, but he already knows his decision, but those with high achievement and scientific excellence suffer from internal conflict and sorrow and pain of a reality that they cannot change. Either in terms of the family that is unable to help him financially or the host country is unable to provide them with university education opportunities or suitable employment opportunities for them. Therefore, in the process of professional decision-making, his choices will not only be based on his abilities, inclinations, or trends but will enter many external factors that will lead to weak decision.

With regard to gender, it is one of the most important factors affecting the individual differences in many things in life, where we find that the physical, mental, and muscular composition of the male differs from the composition of the female which affects the decisions of each, we find that each has interests that suit his composition, and the culture prevailing in society, especially Arab societies, which limit employment opportunities for females and are not allowed to be practiced by females as well as the environment and the community in which they live. The constant and differentiated gender differences in their professional orientations have a significant impact on the identification of the professional environment, and this is due to the amount of information the individual possesses about different professions and their selves, influenced by the religious and ethnic background, the intervention of the family and its aspirations, and the social roles of the females that make them think about choosing careers that are in line with her social life as a mother and worker. The social status and economic level also influence the student's educational and professional decision and cast a shadow over him even if he shows that he is not affected by it.

## 4. Conclusion

This study found a moderate level of professional decision-making skills among tenth-grade refugee students at the University Brigade Institute of the Ministry of Education in Jordan. These students may require specialized training in order to develop better decision-making capacities and to address their challenges with future anxiety, which was found to be high.

The search for predictive ability among these two measures revealed a negative relationship between the two factors. This indicates that professional decision-making helps students to be more comfortable and to reduce negative feelings such as future anxiety.

Furthermore, regarding the gender variable, the results of this study revealed differences between male and female students on their measures of professional decision-making skills and future anxiety: that it was males who were more anxious, and females who demonstrated better decision-making. Thus, future research on these topics should consider gender as a potentially important variable.

## 5. Limitations and Future Directions

This study was conducted with tenth-grade Syrian refugees in public schools at the University Brigade Institute of the Ministry of Education in Jordan. The results of the study were interpreted from the participants' responses to future anxiety and professional decision-making measures. The researchers recommend further research on the professional decision-making skills variable. They also recommend developing training programs for Syrian refugees to teach them decision-making skills to reduce negative psychological effects, including future anxiety, that interfere with the decision-making process. Training should also be developed for counsellors in Syrian refugee schools so that they can help their students develop professional decision-making skills and reduce their future anxiety.

## Figures and Tables

**Table 1:** Averages and standard deviations of professional decision-making and future anxiety.

Variable	Dimension	Mean	Std. deviation	Value
Professional decision-making skill	Flexibility and focus	2.8403	0.61705	Moderate
	Intuition and appreciation	2.8625	1.00441	Moderate
	Authenticity	2.7614	0.96646	Moderate
	Horizon capacity	2.7929	1.97815	Moderate
	Total	2.8270	0.73202	Moderate

Future anxiety		3.5218	0.56114	High

**Table 2:** Averages and standard deviations according to the study variables.

Variable		Future anxiety		Professional decision-making	
		Mean	St. deviation	Mean	St. deviation
Gender	Male	3.7243	0.43736	2.6129	**2.6129**
	Female	2.9783	0.49432	3.4016	**3.4016**
	Total	3.5218	0.56114	2.8270	**2.8270**

Academic achievement	Weak	3.4443	0.52426	3.0688	**3.0688**
	Moderate	3.5111	0.55897	2.8676	**2.8676**
	High	3.5844	0.59034	2.6216	**2.6216**
	Total	3.5218	0.56114	2.8270	**2.8270**

**Table 3:** Results of MANOVA analysis according to the study variables.

Source		Sum of squares	df	Mean square	*F*	Sig.
Gender	Decision-making	16.912	1	16.912	42.685	0.000
	Future anxiety	15.376	1	15.376	74.584	0.000

Academic achievement	Decision-making	3.380	2	1.690	4.266	0.016
	Future anxiety	0.324	2	0.162	0.785	0.458

**Table 4:** Predictive ability of future anxiety, gender, and achievement on professional decision-making skills.

Variable	Model summery		ANOVA		Coefficient		
	*R*	*R* ^2^	*F*	Sig.	Beta	*T*	Sig.
Future anxiety	0.588	0.331	23.959	0.000	-0. 513	15.25	0.000
Gender					0.282	3.269	0.001
Achievement					-0.187	-2.679	0.008

## Data Availability

Data available with order from Ola Al Hwayan by email: o.alhwayan@ju.edu.jo.

## Supplementary Materials

Supplementary Materials The measures: it contains the tools which use in the research The Data file: it includes the data used in extracting research results.
