# Supplementary material for: Predictive Ability of Future Anxiety in Professional Decision-Making Skill among a Syrian Refugee Adolescent in Jordan
Source: Occup Ther Int. 2020 Mar 28;2020:4959785. doi: 10.1155/2020/4959785 (PMC7149486; doi:10.1155/2020/4959785)
Supplement: Supplementary Materials — The measures: it contains the tools which use in the research The Data file: it includes the data used in extracting research results. [file 4959785.f1.zip › measures (1).docx]

**Professional decision-making measure**

| **N** | **Items** | **apply greatly** | **apply highly** | **apply moderately** | **apply simply** | **never apply** |
| --- | --- | --- | --- | --- | --- | --- |
| 1. | **I find it difficult to choose a career.** |  |  |  |  |  |
| 2. | **I keep doing what I do until my choice appears to be a failure.** |  |  |  |  |  |
| 3. | **I suffered from focusing on setting my career goals.** |  |  |  |  |  |
| 4. | **I take my decisions for choosing my studies or career despite the lack of clarity of the goals.** |  |  |  |  |  |
| 5. | **When I make my professional decisions, I expect all the expected possibilities to happen.** |  |  |  |  |  |
| 6. | **I will review my professional decisions if there is a justification.** |  |  |  |  |  |
| 7. | **I use alternative plans if my plans fail or justify it.** |  |  |  |  |  |
| 8. | **I accept any option That I think helps my career choice.** |  |  |  |  |  |
| 9. | **When I have problem choosing my career, I feel like I have new ideas.** |  |  |  |  |  |
| 10 | **I stand at one distance towards all available professional options.** |  |  |  |  |  |
| 11 | **I have the ability to estimate the professional options available.** |  |  |  |  |  |
| 12 | **When I do not reach a suitable professional choice, I wait a while and then come back to make a decision.** |  |  |  |  |  |
| 13 | **I study alternatives before making my professional decision.** |  |  |  |  |  |
| 14 | **I take into account my personal motivations when making my professional decision.** |  |  |  |  |  |
| 15 | **I depend on my self-esteem for taking my decisions when making my professional decision.** |  |  |  |  |  |
| 16 | **I collect enough information before making my professional decision.** |  |  |  |  |  |
| 17 | **I have the ability to find new alternatives to any problem that may confront me in my professional decisions.** |  |  |  |  |  |
| 18 | **I focus on innovative and new results when making my professional decisions.** |  |  |  |  |  |
| 19 | **I tend to take risks and try everything that's new before making professional decisions.** |  |  |  |  |  |
| 20 | **I seek to reach perfect decisions about my professional choice.** |  |  |  |  |  |
| 21 | **I'm trying to find new and unfamiliar alternatives before I make my professional decisions.** |  |  |  |  |  |
| 22 | **I rely on alternatives and ready solutions in my professional decisions.** |  |  |  |  |  |
| 23 | **I try all available means to make the right professional decision.** |  |  |  |  |  |
| 24 | **I consult others before making my professional decision.** |  |  |  |  |  |
| 25 | **I take into account my self-interest only when I make my professional decision.** |  |  |  |  |  |
| 26 | **I think about the future when I make my decision and not only the present.** |  |  |  |  |  |
| 27 | **I accept the information I collect about the profession, even if it contradicts my professional values and ideas.** |  |  |  |  |  |

***Future Anxiety Measure***

| **never apply** | **apply simply** | **apply moderately** | **apply highly** | **apply greatly** | **Items** | **N** |
| --- | --- | --- | --- | --- | --- | --- |
|  |  |  |  |  | **I am deeply concerned by the destruction in my country.** |  |
|  |  |  |  |  | **I am in a state of fear and tension when I think about the future.** |  |
|  |  |  |  |  | **I am disappointed when I think about the future.** |  |
|  |  |  |  |  | **I think my life is going for the worse.** |  |
|  |  |  |  |  | **I have difficulty planning for the future.** |  |
|  |  |  |  |  | **I feel insecure every time I think about the future.** |  |
|  |  |  |  |  | **thinking of my future life makes me unstable.** |  |
|  |  |  |  |  | **My school ambition changed after I left my country.** |  |
|  |  |  |  |  | **I avoid talking to others about the future.** |  |
|  |  |  |  |  | **I am afraid of not having the opportunity to go to the university.** |  |
|  |  |  |  |  | **I have no hope for a better future.** |  |
|  |  |  |  |  | **The best way to live with life is not to think of future.** |  |
|  |  |  |  |  | **I feel the life is meaningless.** |  |
|  |  |  |  |  | **I am disturbed by my overdependence on others.** |  |
|  |  |  |  |  | **I feel weak focus and mind-numbing.** |  |
|  |  |  |  |  | **I have the idea of dying most of** the **time.** |  |
|  |  |  |  |  | **I think I will not be able to achieve my ambitions in the future.** |  |
|  |  |  |  |  | **I fear that future will occur in the future.** |  |
|  |  |  |  |  | **I feel that a future life will be unhappy.** |  |
|  |  |  |  |  | **I'm afraid I don't find a job after graduation.** |  |
|  |  |  |  |  | **I am concerned that** psychological problems will **spreading**  **became of what is now.** |  |
|  |  |  |  |  | **I'm worried about talking about death.** |  |
|  |  |  |  |  | **I'm afraid of losing a member of my family.** |  |
|  |  |  |  |  | **I do not feel calm and comfertable to be away from my country.** |  |
|  |  |  |  |  | **It takes a long time to think and imagine my life in the future.** |  |
|  |  |  |  |  | **I'm afraid of war and the use of weapons.** |  |
|  |  |  |  |  | **I am concerned about the high prices.** |  |
|  |  |  |  |  | **I feel a weak energy and a lack of vital energy.** |  |
|  |  |  |  |  | **I am** concerned that others are **not interested of what is now.** |  |
|  |  |  |  |  | **I have some physical disorders.** |  |
|  |  |  |  |  | **I lack the ability to make a decision.** |  |
|  |  |  |  |  | **I fear that the family will lose its source of income in the future.** |  |
|  |  |  |  |  | **I have a weak motivation to do anything.** |  |
|  |  |  |  |  | **I’m having trouble sleeping.** |  |
